# Decellularized Aged Bruch's Membrane Confers Unique Biochemical Cues to Retinal Pigment Epithelium for In Vitro Modeling of Age‐Related Macular Degeneration

**DOI:** 10.1111/acel.70501

**Published:** 2026-04-24

**Authors:** Blanca Molins, Mercedes Soledad Nabaes Jodar, Eliandre de Oliveira, Josep Maria Estanyol, Esther Peña, Jaione Bezunartea, María Hernández, Marc Figueras‐Roca, Alfredo Adan

**Affiliations:** ^1^ Group of Ocular Inflammation: Clinical and Experimental Studies Institut D'investigacions Biomèdiques Agustí Pi i Sunyer (IDIBAPS) Barcelona Spain; ^2^ Proteomics Laboratory, Biomolecular Analysis Unit. Scientifical and Technological Centers University of Barcelona Barcelona Spain; ^3^ Research Institute Sant Pau (IR SANT PAU) CIBERCV Barcelona Barcelona Spain; ^4^ Retinal Pathologies and New Therapies Group, Experimental Ophthalmology Laboratory, Department of Ophthalmology Clínica Universidad de Navarra Pamplona Spain; ^5^ Navarra Institute for Health Research IdiSNA Pamplona Spain; ^6^ Institut Clínic d'Oftalmologia (ICOF) Hospital Clínic Barcelona Barcelona Spain

**Keywords:** age‐related macular degeneration, Bruch's membrane, decellularized tissue, disease modeling, extracellular matrix, outer blood retinal barrier, proteomics, retinal pigment epithelium

## Abstract

Age‐related macular degeneration (AMD), a chronic inflammatory disease, is a major cause of irreversible blindness worldwide. It involves the degeneration of the retinal pigment epithelium (RPE) and the accumulation of deposits between the RPE and the Bruch's membrane (BrM), ultimately leading to photoreceptor death. The multifactorial and chronic nature of AMD makes it challenging to model in vitro. We developed a biomaterial based on decellularized BrM (dECM‐BrM) from aged donors to evaluate its ability to induce an AMD‐like phenotype in RPE monolayers. BrM from 5 young and 5 aged human donors was decellularized and the protein profile analyzed by LC–MS/MS. dECM‐BrM was then used as a coating substrate for RPE culture. A total of 281 proteins were identified and proteomic analysis screened 49 differentially expressed proteins in aged dECM‐BrM. Gene Ontology analysis showed that they were associated with extracellular region, antioxidant activity, lipid metabolism and transport. Moreover, the KEGG pathway related to complement and coagulation cascade was significantly enriched. RPE culture on aged dECM‐BrM allowed RPE polarization and after 60 days, transepithelial electrical resistance significantly decreased compared to RPE grown on young dECM‐BrM, accompanied by increased IL‐33 secretion and marked expression of drusen components such as vitronectin and apolipoprotein E, lipid deposition and complement factors C3 and C9. We showed a successful approach to obtain a BrM mimic based on decellularized BrM that ensured cellular removal while preserving ECM structure and identified differentially expressed proteins in aged dECM‐BrM that may provide specific biochemical cues fundamental to model AMD in vitro.

## Graphical abstract

A Bruchs membrane (BrM) mimic based on aged decellularized BrM was developed and differentially expressed proteins in aged dECM‐BrM that may provide specific biochemical cues fundamental to model AMD in vitro were identified. RPE culture on aged dECM‐BrM developed certain AMD‐like features including reduced TEER and expression of drusen components.

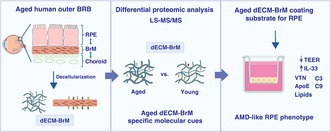

## 1 Introduction

Age‐related macular degeneration (AMD) is a devastating, chronic, inflammatory disease that represents the main cause of irreversible blindness among people above 65 worldwide (Wong et al. 2014). It is characterized by loss of central vision due to degeneration of the macula, the small central region of the retina responsible for fine vision. The retinal pigment epithelium (RPE), together with the Bruch's membrane (BrM) and the choriocapillaris, integrates the outer blood retinal barrier (oBRB) and is the prime target of AMD. The RPE monolayer, strategically placed below the photoreceptors, is fundamental in preserving ocular function as it is the only metabolic supply of the outer retina and is responsible for the phagocytosis of the photoreceptor outer segments. The RPE also regulates light absorption and molecular transport, recycles visual cycle components, and preserves the retinal immune privilege (Strauss 2005). The basolateral side of the RPE lies on the BrM, the acellular membrane rich in collagen and elastin that controls the exchange of biomolecules between the choriocapillaris and the RPE (Booij et al. 2010).

Early and intermediate AMD stages are characterized by the accumulation of drusen, deposits of extracellular matrix (ECM), lipids, minerals, and other proinflammatory components beneath the RPE. Disease progression can lead either to neovascular AMD (nAMD), due to aberrant outgrowth of neovessels across the BrM and beneath the RPE, or to geographic atrophy (GA) that results in photoreceptor and RPE death, both forms resulting in irreversible vision loss. Whereas nAMD can be efficiently managed with intravitreal injections of anti‐angiogenic agents, until recently the only approved therapy for GA was the use of dietary vitamins (AREDS). In 2023, the FDA, but not the EMA, approved the use of pegcetacoplan and avançincaptad pegol, regulators of the complement mediators C3 and C5, respectively, for the treatment of GA (Tzoumas et al. 2023). Yet, the therapeutic benefits of such therapies have shown limited efficacy, urging the development of novel therapeutic approaches.

Several environmental factors –smoking, diet, western lifestyle‐ and genetic factors, especially complement‐related genes, have been linked to the development of AMD, but advanced age stands as the strongest risk factor for AMD (Lambert et al. 2016). Although the exact mechanisms are still being unraveled, age‐related alterations such as inflammaging –chronic inflammation–, senescence, altered lipid and energy metabolism, increased oxidative stress, and complement activation in the RPE and choriocapillaris may lead to AMD in susceptible individuals (Nussenblatt et al. 2014; Romero‐vazquez et al. 2021). Besides the impact on RPE function, with increasing age, the capacity of the BrM to facilitate macromolecular exchange between the choroidal and RPE compartments is compromised due to increased thickness, collagen crosslinking and reduced elasticity that may further promote drusen formation and RPE dysfunction (Curcio et al. 2011).

The complex and multifactorial nature of AMD combining genetic and environmental factors renders the development of adequate AMD models highly challenging. The use of murine models has been very useful to interrogate certain features of AMD, such as the contribution of immune cells and oxidative stress (Beguier et al. 2020; Soundara Pandi et al. 2021) but the different regulation of the innate immune system and the lack of macula in these animals make them unsuitable for properly replicating human disease. Although non‐human primates do possess a cone‐rich macula, ethical and cost concerns make them also unsuitable. In addition, advanced age, the main contributor to AMD, is extremely difficult to replicate in vivo with animal models with a short lifespan. On the other hand, in vitro models of AMD using primary RPE cultures grown on filters have been able to replicate drusen‐like formation and other features of RPE dysfunction (Johnson et al. 2001; Shaw et al. 2024). The advent of stem cell technologies and biomaterial engineering is offering novel tools to develop more physiologically relevant models of AMD. Besides the use of adequate RPE cells, replicating the BrM is also key to properly recapitulate AMD‐related changes in vitro. The development of materials to recapitulate the BrM has been mostly motivated by the need for suitable BrM substitutes for RPE transplantation, and a variety of synthetic, naturally‐derived, and hybrid materials have been developed to mimic the BrM (Murphy et al. 2020; Molins et al. 2024). In this regard, decellularized extracellular matrix‐ (dECM)‐based biomaterials offer a step forward in reproducing the native tissue as they preserve tissue ultrastructure and composition, providing a complex site‐specific combination of biochemical and biophysical cues (Badylak et al. 2011). Of note, several groups have engineered different types of biomaterials based on decellularized ocular tissue such as retina, uvea, sclera, and BrM to study RPE engraftment, function, and differentiation (Li et al. 2022; Maqueda et al. 2021; Chirco et al. 2017; Kim et al. 2021).

In the present study, we hypothesize that the ECM from aged oBRB may provide unique biochemical cues that can induce an AMD‐like phenotype in the RPE that can be implemented for AMD modeling. For this purpose, we developed a biomaterial based on decellularized RPE/BrM/choroid (dECM‐BrM) from aged human donors and compared its proteomic profile to dECM‐BrM obtained from young donors. We then used aged dECM‐BrM as a coating substrate for primary RPE culture and evaluated its impact on RPE phenotype.

## 2 Methods

### 2.1 Tissue Collection and Processing

Human donor eyes were obtained from the Banc de Sang i Teixits (Barcelona, Spain). Research was performed in accordance with the Declaration of Helsinki and the Hospital Clínic of Barcelona Institutional Review Board (IRB) approved this study according to local and national IRB guidelines (HCB/2022/0089). Ten aged (> 65 years old) and 10 young (< 45 years old) cadaveric eyes with no history of retinal disease were used (Table 1). Eyes were enucleated within 8 h after death and stored at −80°C until used for experiments. Eyes were thawed overnight at 4°C, then the eyeball was hemisected to remove the anterior segment and the vitreous humor. The hemisected eye‐cup was flooded with phosphate buffer saline (PBS) and the retina peeled gently from the RPE. The RPE was scraped out and BrM/choroid was peeled from the sclera.

**TABLE 1:** Donor globe characteristics.

Donor	Age	Sex	Cause of death	Assay
1	45	Male	Hemmorhagic stroke	b, c
2	43	Male	Cardiovascular disease	a, c
3	32	Female	Encephalic edema	b, c
4	20	Female	Sudden death	a, b
5	42	Male	Hemorrhagic stroke	a, b
6	69	Male	Hemorrhagic stroke	a, b
7	66	Female	Myocardial infarction	a, c
8	67	Male	Cardiovascular disease	b, c
9	69	Male	Hemorrhagic stroke	a, b
10	65	Female	Hemorrhagic stroke	b, c

*Note:* ^a^Biochemical characterization of native and decellularized ECM‐BrM, ^b^Differential proteomics, ^c^dECM‐BrM coating biomaterial.

Porcine eyes obtained from a local abattoir were also used for the initial experiments to establish the optimal experimental conditions for the decellularization protocol, pepsin digestion conditions, and proteomics analysis.

### 2.2 Decellularization of the RPE/BrM/Choroid Complex

Decellularization of the RPE/BrM/choroid tissue was based on our established protocol (Molins et al. 2021) with some modifications to maximize cell removal and ECM preservation. Trypsin/EDTA 0.05% was applied for 3 h at 37°C, then PBS was applied for 15 min to rinse the tissue. Afterwards, 1% sodium dodecyl sulfate (SDS) was added for 30 h, changing the solution twice per day with quick rinses in 1% SDS. Then ddH_2_O was applied for 48 h, changing solution twice per day with quick rinses with ddH_2_O, and finally, PBS containing 1% of penicillin–streptomycin was applied for 8 h.

### 2.3 Second Harmonic Generation (SHG) and Two‐Photon Excited Fluorescence Microscopy (TPEF)

Decellularized and native RPE/BrM/choroidal tissues were imaged by a nonlinear technique of SHG and TPEF that enables visualization of collagen and elastin in unstained tissues (Garreta et al. 2016). As a non‐centrosymmetric molecule, collagen can generate the second harmonic of incident light, while elastin is a significant source of ECM autofluorescence that can be imaged by TPEF. The SHG setup consisted of a Leica inverted confocal laser scanning microscope SP‐5 with META scanning module equipped with a mode‐locked near infrared MAITAI Wide Band (710 nm–990 nm) laser. The exciting laser beam was tuned to 810 nm and the SHG collagen signal was obtained using a 447–453 nm bandpass filter. DAPI was used to stain nuclei and images were taken using a water 25× objective.

### 2.4 Biochemical Characterization

DNA was extracted from native and dECM‐BrM digests according to the manufacturer's protocol of the DNeasy Blood & Tissue kit (QIAGEN, Netherlands) and DNA concentration was measured using a Nano Drop 2000 (MaestroNano, USA). Elastin was extracted with 0.25 *M* oxalic acid at 100°C, then centrifuged at 10,000 *g* and the supernatant saved. Elastin was then determined using the Fastin Elastin kit (Biocolor, Belfast, N. Ireland) according to the manufacturer's instructions. Soluble collagen in native and decellularized samples was determined using the Sircol 2.0 Soluble Collagen Assay Kit (Biocolor, Belfast, N. Ireland) according to the manufacturer's instructions.

### 2.5 Lyophilization and Digestion of dECM‐BrM for Coating Preparation

Decellularized samples were then frozen at −80°C overnight and subjected to lyophilization using a freeze drier with the temperature maintained within the sample chamber (−50°C to −60°C) and air dried for 48–72 h. Samples were then finely cut and digested with pepsin‐HCl (1 mg pepsin in 0.01 N HCl per 10 mg of dECM‐BrM) for 3 days at room temperature under constant stirring. The digested tissue was neutralized with 10× PBS and 0.1 N NaOH and centrifuged at 3000 rpm for 15 min and filtered. The resulting dECM‐BrM solution was stored at −20°C or at 4°C for 2 months maximum until further use.

### 2.6 Protein Sample Preparation and Liquid Chromatography With Tandem Mass Spectrometry (LC–MS/MS) Analysis

Lyophilized dECM‐BrM from 4 aged and 4 young donors were lysed with 1 mL RIPA Lysis buffer and incubated for 30 min on ice, then frozen at −80°C overnight and centrifuged at 14000× *g* for 15 min at 4°C. The supernatant was collected and the protein concentration was measured using the BCA protein assay. dECM‐BrM protein samples (10 μg) were mixed with loading buffer and boiled at 90°C for 5 min prior to loading on 6% stacking SDS‐PAGE. Once samples entered the stacking gel, electrophoresis was stopped, and protein bands were carefully excised from the gel. Gel pieces were then washed and fixed in 10% acetic acid, 40% ethanol for 1 h under mild agitation. Afterwards, gels were washed with ddH_2_O thrice.

Samples were in‐gel digested as follows: Gel bands were washed with ammonium bicarbonate (50 mM NH_4_HCO_3_) and acetonitrile (ACN), reduced with dithiothreitol (20 mM) for 60 min at 60°C, and alkylated with iodoacetamide (55 mM) for 30 min at 25°C in the dark. Afterwards, samples were overnight digested with trypsin at 37°C. Finally, the resulting peptide mixtures were extracted from the gel matrix with 50 mM NH_4_HCO_3_ in 50% ACN and 100% ACN, and dried down in SpeedVac vacuum system and stored at −20°C until LC–MS analysis.

The dried‐down peptide mixture was analyzed in a nanoAcquity liquid chromatographer (Waters) coupled to a LTQ‐Orbitrap Velos (Thermo Scientific) mass spectrometer. The tryptic digests were resuspended in 1% formic acid solution and an aliquot was injected for chromatographic separation three times. Peptides were trapped on a Symmetry C18 trap column and separated using a C18 reverse phase capillary column.

Eluted peptides were subjected to electrospray ionization in an emitter needle with an applied voltage of 2000 V. Three different peptide masses ranges (m/z 300–600, m/z 580–780, and m/z 750–1600) were analyzed in data dependent mode where a full Scan MS was acquired in the Orbitrap with a resolution of 60,000 FWHM at 400 m/z. Up to the 15th most abundant peptides (minimum intensity of 500 counts) were selected from each MS scan and then fragmented in the linear ion trap using CID (38% normalized collision energy) with helium as the collision gas. Ions selected for MS/MS fragmentation were dynamically excluded for 30 s. The scan time settings were: Full MS: 250 ms (1 microscan) and MSn: 120 ms. Generated .raw data files were collected with Thremo Xcalibur (V.2.2).

### 2.7 Proteomics Bioinformatic Analysis: Database Search and Label‐Free Quantification

The .raw datafiles obtained in the mass spectrometry analyses were used to search against the public database SwissProt human. Database search was performed with SequestHT search engine using Thermo Proteome Discover (v.2.5). The following search parameters were applied: carbamidomethylation of cysteines as fixed modifications; methionine oxidation and acetylation, met‐loss, and met‐loss‐acetylation as variable modifications. The enzyme was trypsin, with a maximum allowed missed cleavage of 2. For label‐free quantification (LFQ), the minimum ratio count was set to 2, and unique peptides were used for quantification. The false discovery rate (FDR) was set to 1% for peptide spectrum match levels.

Intensity‐based absolute quantification (iBAQ) values were determined with MaxQuant software and relative iBAQ (riBAQ) was obtained by dividing iBAQ values of each protein with the sum of iBAQ in each sample for comparison across different samples. Matrisome AnalyzeR was used to cluster all detected proteins into matrisome‐related subgroups (Petrov et al. 2023). Matrisome contents related to the structural protein components were termed as “core matrisome”, including collagens, glycoproteins, and proteoglycans. The rest were defined as “matrisome‐associated proteins” and included ECM regulators, ECM‐affiliated proteins, and secreted factors.

LFQ values that represent normalized peptide intensities were used for screening the differentially expressed proteins between aged and young dECM‐BrM (fold change > 1.5 and *p* < 0.05). The Database for Annotation, Visualization, and Integrated Discovery (DAVID) was selected to analyze Gene Ontology (GO) biological domains including Biological Process (BP), Molecular Function (MF), Cellular Component (CC), and Kyoto Encyclopedia of Genes and Genomes (KEGG) pathways (https://davidbioinformatics.nih.gov) (Sherman et al. 2022; Huang et al. 2009). The Benjamini–Hochberg procedure was used to control the false discovery rate at *p* < 0.05. The STRING database was used to construct the protein–protein interactions (PPIs) network (version 12.0, https://cn.string‐db.org/) of the differentially abundant proteins with organism set to *Homo sapiens* and confidence medium (0.4).

### 2.8 Primary RPE Cell Culture

#### 2.8.1 Porcine RPE

Primary porcine RPE cells were isolated and cultured following the protocol described by Toops with some modifications (Toops et al. 2014). Porcine eyes collected fresh from a local abattoir within 4 h after slaughter were trimmed of excess tissue and placed in 0.2% povidone iodine for 10 min on ice. Eyes were rinsed with sterile distilled water and placed in 1000 U/mL Penicillin–Streptomycin on ice for a minimum of 5 min. Anterior segments were removed with a scalpel at the ora serrata. Eyecups were filled with 1 mM EDTA and incubated at 37°C for 30 min to loosen the neural retina from the RPE sheet. The retina was gently pulled and detached from the RPE sheet. RPE cells were collected after 30 min incubation of the eyecups with 0.05% trypsin with 0.67 mM EDTA at 37°C. After trypsin inactivation with 10% FBS, RPE suspension was centrifuged and plated in DMEM High Glucose (Capricorn Scientific), with L‐glutamine and sodium pyruvate, supplemented with 1% penicillin–streptomycin, 1% non‐essential amino acids (Corning) and 10% FBS. At Day 3 of culture, 5 μg/mL of ciprofloxacin (Sigma) was added to the medium and at Day 7, serum was decreased to 1%. Cell monolayers were pigmented and showed the characteristic cobblestone morphology. At Day 14, RPE cells were trypsinized and plated at confluence onto semi‐permeable polycarbonate Transwell filters, 0.4 μm pore size, previously coated with aged or young dECM‐BrM (100 μg/mL). RPE cells were maintained in a 37°C and 5% CO_2_ incubator for up to 15 weeks and fed with 1% FBS growth medium every 3–4 days.

#### 2.8.2 Human RPE

Primary adult human RPE (hRPE) cells were isolated from two human donor eyes (donor ages: 67 and 77). Donations were acquired through the Department of Pathology, Anatomy and Physiology of the School of Medicine, University of Navarra (ethics committee 025.072) as previously described (González‐Zamora et al. 2022). Globes were immersed in 10% povidone iodine for 2 min and then cut below the ora serrata to separate the posterior eyecup. The neural retina was peeled out and RPE cells were softly dislodged by brushing with a fire‐polished glass spatula. hRPE cells were centrifuged at 200 *g* for 10 min, and the pellet was suspended in DMEM:F‐12 medium supplemented with 10% FBS and 1% penicillin/streptomycin, plated into 24‐well tissue culture plates, and grown to confluence in a standard incubator at 37°C under humidified 5% CO_2_ conditions. After proliferation, hRPE cells were trypsinized and cryopreserved until use. Thawed cells from each donor were seeded in 24‐well tissue culture plates previously coated with aged or young dECM‐BrM (100 μg/mL). hRPE cells were maintained for up to 9 weeks and fed with 1% FBS DMEM:F‐12 growth medium every 3–4 days. Triplicates of each donor and passages 1 to 3 were used in the study.

### 2.9 Measurement of Transepithelial Electrical Resistance (TEER)

TEER was measured using a commercial electrical resistance system (Millicell; Millipore) in primary RPE monolayers grown on Transwell filters as described above. TEER values were calculated by subtracting the value of a blank (Transwell filter without cells). Measurements were repeated at least three times for each filter, and each experiment was repeated at least three times using 2 filters.

### 2.10 Immunofluorescence

After three washes with PBS, RPE grown on inserts were fixed with 4% paraformaldehyde for 20 min at room temperature and washed three times with PBS. For ZO‐1 immunostaining, cells were permeabilized with 0.2% Triton X‐100 for 15 min. Samples were then blocked using PBS with 1% bovine serum albumin (BSA) for 30 min at room temperature and incubated overnight at 4°C with primary antibodies diluted in PBS with 1% BSA. The following antibodies were used: ZO‐1 (clone 1A12, Invitrogen), vitronectin (15833–1‐AP, Proteintech), Apolipoprotein E (PA5‐27088, Invitrogen), complement C3 (PA1‐29715, Invitrogen), complement Factor H (OX‐24, Abcam), complement C9 (PA5‐29093, Invitrogen), collagen IV (PAI28534, Invitrogen). After three washes with PBS, samples were treated for 1 h at room temperature with fluorescent‐conjugated secondary antibodies. Nile Red (N1142, Invitrogen) was used to visualize neutral lipids. Nuclei were detected using 4,6‐diamidino‐2‐phenylindole (DAPI; 1:5000, D1306, Life Technologies) for 10 min. For mounting, samples were immersed in Prolong Gold Antifade reagent (Invitrogen). Image acquisition was carried out using an SP5 Leica inverted microscope confocal laser microscope. Fluorescent images were acquired in a spatial data set (z‐sections). Image analysis was done in Fiji (ImageJ, v1.53t; National Institutes of Health, Bethesda, MD, USA). Mean fluorescence intensity was determined in z‐stacks (sum projections) normalized to nuclei count. Data were obtained from at least four confocal z‐stacks of each sample from duplicates of three independent pRPE experiments and two independent hRPE experiments.

### 2.11 Enzyme‐Linked Immunosorbent Assay

Supernatants from apical and basolateral compartments of RPE cells were collected and centrifuged (1000 *g* for 5 min) to remove particulates and stored at −70°C until further analysis. Interleukin (IL)‐8, IL‐33, IL‐17, IL‐18, and tumor necrosis factor‐α (TNF‐α) were measured using human DuoSet ELISA development kits (R&D Systems).

## 3 Results

### 3.1 Preparation and Characterization of Decellularized ECM‐BrM

**FIGURE 1:**
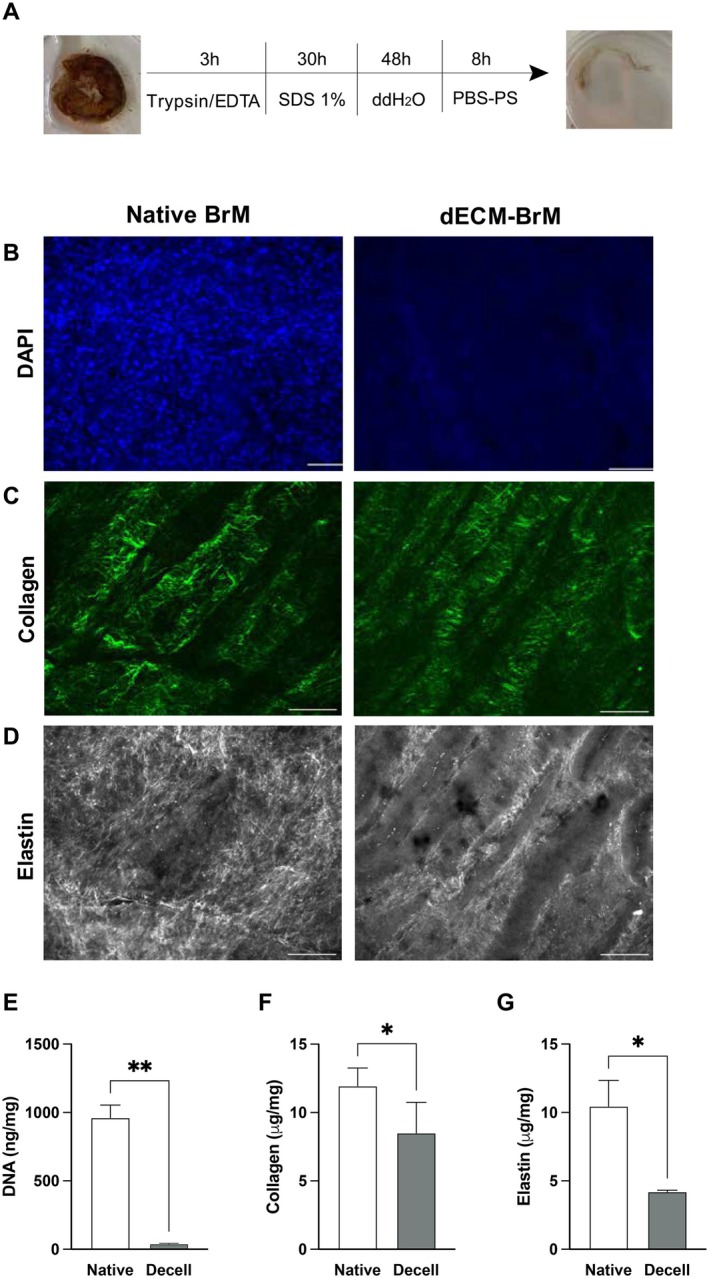
Preparation and characterization of human dECM‐BrM. (A) Schematic representation of the decellularization process. (B) DAPI staining of native and decellularized human BrM. (C) Second harmonic generation (SHG) 2‐photon microscopy showing the presence of collagen fibers in native and dECM‐BrM. (D) TPEF showing elastin fibers in native and dECM‐BrM. Scale bar: 100 μm. Biochemical composition of the native and dECM‐BrM isolated from porcine eyes: (E) host DNA (ng/mg), (F) Soluble collagen I content (μg/mg), and (G) Elastin content (μg/mg). Data are shown as mean ± SEM, *n* = 3. Statistical analysis was performed by Student's *t*‐test. *p < 0.05, **p < 0.001.

The RPE/BrM/choroid complex was decellularized with an optimized protocol based on our previous work (Molins et al. 2021). To better preserve the ECM while ensuring cellular removal, we applied some modifications to our previous protocol (Figure 1A). Treatment with Trypsin/EDTA for 3 h, followed by 30 h treatment with SDS 1%, 48 h ddH_2_O and a final 8 h rinse with PBS resulted in efficient cellular removal as DNA content was below 50 ng/mg and no nuclei were observed by DAPI staining (*p* < 0.05) (Figure 1B,E). Noteworthy, collagen fibers were largely preserved in decellularized samples, as seen by SHG microscopy (Figure 1C). Quantification of soluble collagen showed a≈20% reduction in decellularized tissue (*p* < 0.05) (Figure 1F). Instead, TPEF revealed a notable disruption in elastin distribution in decellularized samples compared to native BrM (Figure 1D). Indeed, elastin quantification also showed a significant reduction of more than 50% in decellularized samples (*p* < 0.05) (Figure 1G).

### 3.2 Proteomic Analysis Revealed Preservation of the ECM in dECM‐BrM

**FIGURE 2:**
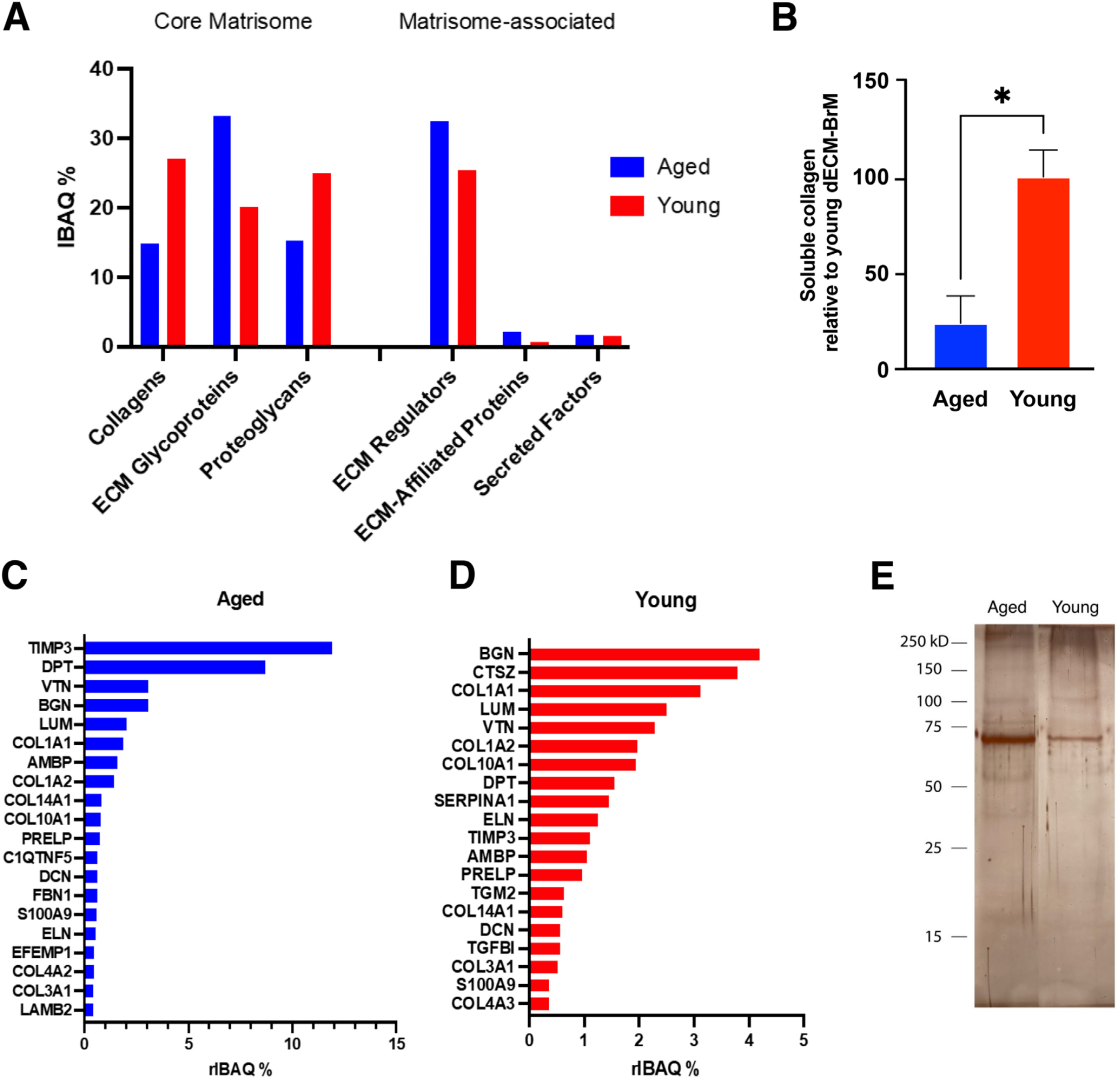
Proteomic analysis of young and aged human dECM‐BrM. (A) Percent distribution of matrisome‐related proteins for aged and human dECM‐BrM. (B) Content of soluble collagen in young and aged dECM‐BrM (*n* = 3), *p < 0.05 calculated using Student's *t*‐test. The top 20 most abundant matrisome proteins in aged (C) and young (D) dECM‐BrM (*n* = 4). (E) Silver staining of SDS‐PAGE gels of aged and young dECM‐BrM.

To characterize the proteomics content after decellularization, mass spectrometry was performed on lyophilized dECM‐BrM extracts from 4 young and 4 aged donors. A total of 399 proteins were identified (FDR < 5%) at protein level by Proteome Discoverer (v 2.5). After excluding proteins that did not have peptides identified in at least two samples, 281 proteins remained (see Table S1). The identified matrisome proteins (around 50% of total identified proteins) were categorized as core matrisome and matrisome‐associated proteins based on the Matrisome database and published reports (Naba et al. 2012). Among the core matrisome, ECM glycoproteins were the most abundant subcategory in aged dECM whereas in young dECM‐BrM collagens were the most abundant (Figure 2A). Indeed, soluble collagen in young dECM‐BrM was 4 times higher than in aged dECM‐BrM (*p* < 0.05) that could reflect a higher level of insoluble collagen due to increased crosslinking in aged samples (Figure 2B). Both young and aged dECM‐BrM contained significant amounts of matrisome‐associated proteins, especially ECM regulators in both cases. The top 20 matrisome proteins with the highest riBAQ were noted (Figure 2C,D). Both young and aged dECM‐BrM were enriched in collagen I and proteoglycans (biglycan [BGN] and lumican [LUM]). Aged dECM‐BrM was particularly enriched in glycoproteins (dermatopontin [DPT] and vitronectin [VTN]) and ECM regulators (metalloprotease inhibitor 3 [TIMP3] and AMBP protein). The top 3 most abundant categorical matrisome proteins containing full name and abbreviation are listed in Table 2. The remaining non‐matrisome identified proteins included serum amyloid P component, serum albumin, apolipoproteins, clusterin, complement factors, among others as shown in Tables 3 and 4. To identify protein categories significantly enriched in dECM‐BrM proteome, mass spectrometry data were analyzed using DAVID bioinformatic tool. Gene ontology biological process (GOBP) enrichment analysis revealed that dECM‐BrM proteome was significantly enriched in processes related to “cell adhesion”, “proteolysis”, “angiogenesis”, “ECM organization”, “inflammatory response”, “innate immune response”, “complement activation”, and “collagen organization” among others (Table S2). In the cellular component analysis, dECM‐BrM proteome was, as expected, mainly enriched in “extracellular region”, “matrix‐related components”, and “membrane and cell surface structures” (Table S3). The results of molecular function and KEGG analysis are shown in Tables S4 and S5.

**TABLE 2:** Top matrisome proteins in young and aged dECM‐BrM.

	Young		Aged	
	Gene symbol	Protein	Gene symbol	Protein
*Core matrisome*				
Collagens	COL1A1	Collagen α‐1(I) chain	COL1A1	Collagen α‐1(I) chain
	COL1A2	Collagen α‐2(I) chain	COL1A2	Collagen α‐2(I) chain
	COL10A1	Collagen α‐1(X) chain	COL14A1	Collagen α‐1(XIV) chain
ECM Glycoproteins	VTN	Vitronectin	DPT	Dermatopontin
	DPT	Dermatopontin	VTN	Vitronectin
	ELN	Elastin	FBN1	Fibrillin1
Proteoglycans	BGN	Biglycan	BGN	Biglycan
	LUM	Lumican	LUM	Lumican
	PRELP	Prolargin	PRELP	Prolargin
*Matrisome‐associated*				
ECM Regulators	CTSZ	Cathepsin Z	TIMP3	Metalloproteinase inhibitor 3
	SERPINA1	α‐1‐antitrypsin	AMBP	α‐1‐microglobulin
	TIMP3	Metalloproteinase inhibitor 3	SERPINA1	α‐1‐antitrypsin
ECM‐affiliated proteins	LGALS1	Galectin‐1	C1QTNF5	Complement C1q tumor necrosis factor‐related protein 5
	ANXA1	Annexin A1	LGALS1	Galectin‐1
	ANXA5	Annexin A5	ANXA5	Annexin A5
Secreted Factors	S100A9	Protein S100‐A9	S100A9	Protein S100‐A9
	FLG2	Filaggrin‐2	CXCL12	Stromal cell‐derived factor 1
	HRNR	Hornerin	FLG2	Filaggrin‐2

**TABLE 3:** Top 10 non‐matrisome proteins in young dECM‐BrM.

Young		
Gene symbol	Protein	RIBAQ%
APCS	Serum amyloid P‐component	13.49
HIST1H4A	Histone H4	8.90
PRSS2	Putative trypsin‐6	8.60
ALB	Serum albumin	6.50
HBB	Hemoglobin subunit beta	4.93
DEFA3	Neutrophil defensin 3	4.54
HIST2H2BE	Histone H2B type 2‐E	2.20
AHSG	Alpha‐2‐HS‐glycoprotein	1.80
HIST1H2BL	Histone H2B type 1‐L	1.30
LYZ	Lysozyme C	1.26

**TABLE 4:** Top 10 non‐matrisome proteins in aged dECM‐BrM.

Aged		
Gene symbol	Protein	RIBAQ%
ALB	Serum albumin	8.77
APCS	Serum amyloid P‐component	8.37
HIST1H4A	Histone H4	7.76
APOE	Apolipoprotein E	6.96
HBB	Hemoglobin subunit beta	3.10
PRSS2	Putative trypsin‐6	2.80
CLU	Clusterin	2.65
DEFA3	Neutrophil defensin 3	2.43
C9	Complement component C9	1.59
LYZ	Lysozyme C	1.52

### 3.3 Differential Proteomics Analysis of Young and Aged dECM‐BrM

**FIGURE 3:**
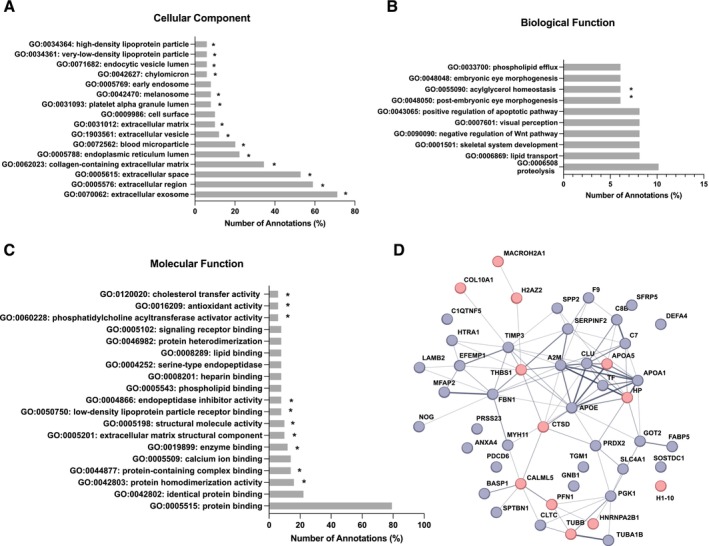
Proteomic analysis of the screened differentially expressed proteins in young versus aged dECM‐BrM. Gene ontology (GO) enrichment analysis of differentially expressed proteins in young versus aged dECM‐BrM. (A) Cellular component, (B) Biological process, and (C) Molecular function. (D) Protein protein interaction (PPI) network analysis of the screened differential genes. Line thickness indicates strength of data support.

To obtain a comprehensive picture of aged and young dECM‐BrM proteomes, silver staining of SDS‐PAGE and differential mass spectrometry was performed in aged and young dECM‐BrM protein extracts. Silver staining showed an overall reduction in the number and intensity of protein bands in young dECM‐BrM compared to aged samples (Figure 2E). Then, differential proteomic analysis of aged and young dECM‐BrM revealed relatively high abundance in 37 proteins in aged dECM‐BrM and 12 proteins in young dECM‐BrM samples with fold change greater than 1.5 between each other and *p* < 0.05 (Tables 5 and 6). Of note, there were 7 proteins present in aged dECM‐BrM that were not detected in young dECM‐BrM. To identify protein categories significantly enriched in differentially expressed proteins in aged and young dECM‐BrM, mass spectrometry data was analyzed using the DAVID bioinformatic tool. In GO cellular component (CC) analysis, differentially expressed proteins were significantly enriched in 14 categories including “extracellular exosomes”, “collagen‐containing extracellular matrix”, “extracellular space”, “extracellular vesicle”, “melanosome”, or “extracellular matrix” (*p* < 0.05) (Figure 3A). In terms of biological process, differentially expressed proteins were significantly enriched in “post‐embryonic eye morphogenesis” and “acylglycerol homeostasis” (*p* < 0.05, Figure 3B) and regarding molecular function, differentially expressed proteins were significantly enriched in 10 categories (*p* < 0.05) (Figure 3C). The KEGG pathway analysis showed that the differentially expressed proteins set was significantly enriched in “complement and coagulation cascade pathway” (*p* = 0.0023).

**TABLE 5:** Screened highly abundant proteins in aged dECM‐BrM.

Gene symbol	Protein description	Fold‐change	*p*
DEFA4	Defensin alpha 4	100	NA
GOT2	Aspartate aminotransferase	100	NA
ANXA4	Annexin A4	100	NA
F9	Coagulation factor IX	100	NA
SFRP5	Secreted frizzled‐related protein 5	100	NA
IGKV3‐20	Immunoglobulin kappa variable 3–20	100	NA
FABP5	Fatty acid binding protein 5	100	NA
C1QTNF5	Complement C1q tumor necrosis factor‐related protein 5	18.184	0.039
TIMP3	Metalloproteinase inhibitor 3	16.81	0.015
SLC4A1	Band 3 anion transporter protein	13.02	0.007
EFEMP1	EGF‐containing fibulin‐like extracellular matrix protein 1	12.97	0.042
PRSS23	Serine protease 23	11.26	0.019
FBN1	Fibrillin‐1	8.84	0.003
SOSTDC1	Sclerostin domain‐containing protein 1	8.66	0.027
APOE	Apolipoprotein E	8.11	0.001
CLTC	Clathrin heavy chain 1	7.23	0.046
SERPINF2	Alpha‐2‐antiplasmin	6.74	0.024
HTRA1	Serine protease HTRA1	6.73	0.001
CLU	Clusterin	6.56	0.006
LAMB2	Laminin subunit beta‐2	5.09	0.043
APOA1	Apolipoprotein A‐1	4.81	0.002
C7	Complement component C7	4.41	0.034
BASP1	Brain acid soluble protein 1	3.86	0.000
C8B	Complement component C8 beta chain	3.82	0.045
MFAP2	Microfibrillar‐associated protein 2	3.65	0.049
PGK1	Phosphoglycerate kinase 1	3.60	0.000
PDCD6	Programmed cell death protein 6	3.33	0.046
SPP2	Secreted phosphoprotein 24	3.04	0.030
TF	Serotransferrin	2.91	0.021
GNB1	Guanine nucleotide‐binding protein G(I)/G(S)/G(T) subunit beta‐1	2.71	0.009
TUBA1B	Tubulin alpha‐1B chain	2.70	0.025
A2M	Alpha‐2‐macroglobulin	2.39	0.019
NOG	Noggin	2.13	0.028
MYH11	Myosin‐11	2.06	0.026
SPTBN1	Spectrin beta chain	1.86	0.002
TGM1	Protein‐glutamine gamma‐glutamyltransferase	1.83	0.044
PRDX2	Peroxiredoxin‐2	1.71	0.040

**TABLE 6:** Screened highly abundant proteins in young dECM‐BrM.

Gene symbol	Protein description	Fold change	*p*
MACROH2A1	Core histone macro‐H2A.1	9.52	0.012
H2AZ2	Histone H2A.V	7.69	0.012
HI‐10	Histone H1.10	5.56	0.010
CALML5	Calmodulin‐like protein 5	4.81	0.001
HP	Haptoglobin	4.67	0.008
COL10A1	Collagen alpha‐1(X) chain	4.29	0.040
HNRNPA2B1	Heterogeneous nuclear ribonucleoproteins A2/B1	2.80	0.011
THBS1	Thrombospondin‐1	2.45	0.043
CTSD	Cathepsin D	2.40	0.009
TUBB	Tubulin beta chain	2.10	0.019
PFN1	Profilin‐1	1.78	0.019
APOA5	Apolipoprotein A‐V	1.50	0.005

PPI network analysis of the differentially abundant proteins was performed using STRING, which integrates interaction data from several sources with information on physical and functional properties and with known and predicted protein interactions (Figure 3D). Functional PPI network included 48 nodes and 95 interactions (edges), with an average local clustering coefficient of 0.463, an average node degree of 3.96, and PPI enrichment *P* < 1.0E^−16^. Strong PPIs were clustered around APOE, APOA1, APOA5, CLU, C7, and C8.

### 3.4 Aged dECM‐BrM Based Substrate Supports Primary RPE Culture Promoting AMD‐Associated Phenotypic Features

**FIGURE 4:**
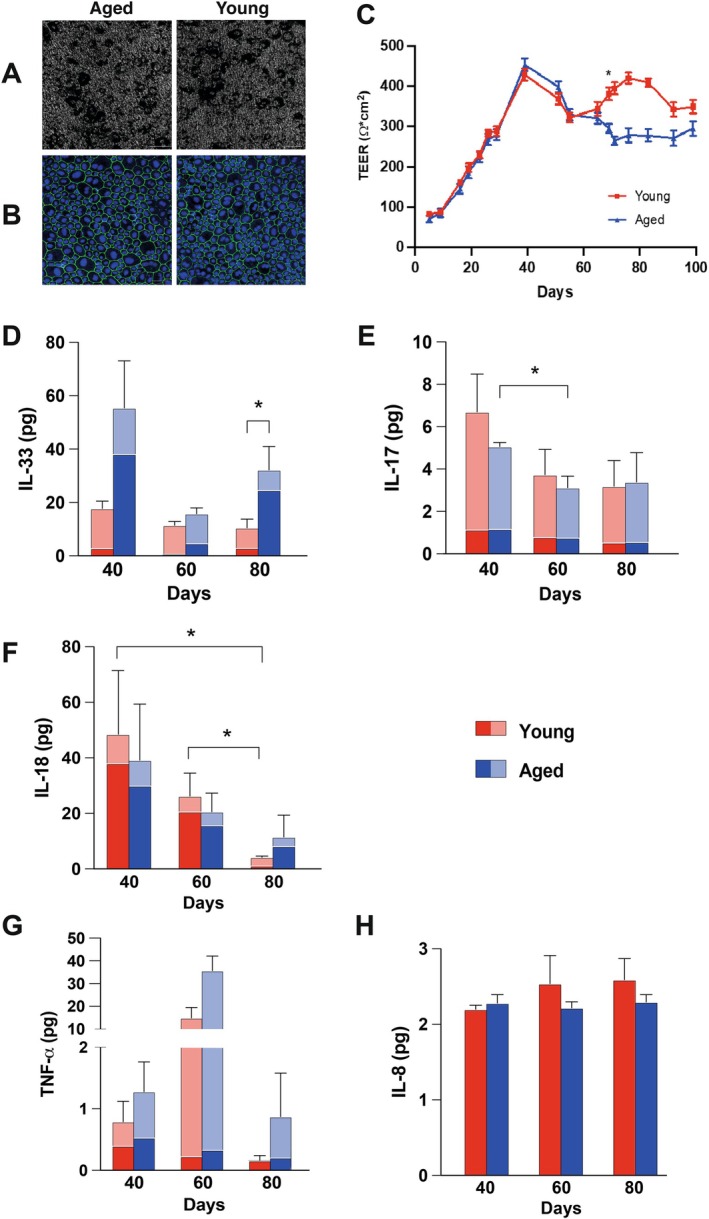
Effect of aged dECM‐BrM coating substrate on pRPE function and phenotype. (A) Confocal microscopy images of transmitted light showing cobblestone morphology and pigmentation of pRPE cells grown on aged and young dECM‐BrM. (B) Immunofluorescence of ZO‐1 staining (green) of pRPE cells grown on Transwell inserts for 100 days on aged and young dECM‐BrM. Scale bar = 50 μm. (C) Transepithelial electrical resistance (TEER) of primary pRPE cells grown on Transwell inserts coated with aged or young dECM‐BrM for up to 100 days. Data are shown as mean ± SEM, *n* = 3. Statistical analysis was performed by Student's *t*‐test. *p < 0.05. Secretion of IL‐33 (D), IL‐18 (E), IL‐17 (F), TNF‐α (G), and IL‐8 (H) by pRPE cells grown on dECM‐BrM‐coated transwell inserts. Light color indicates basolateral secretion whereas dark color reflects apical secretion. Data are shown as mean ± SEM, *n* = 3. *p < 0.05 calculated using Mann–Whitney for comparisons between two independent conditions, and Kruskal‐Wallis followed by post hoc Dunn's test for comparisons across multiple time points.

Proteomics analysis not only showed that dECM‐BrM preserved ECM components but also revealed a differential protein profile of aged dECM‐BrM. We hypothesized that a coating substrate based on dECM‐BrM would allow RPE survival and that aged dECM‐BrM would provide unique biochemical cues to induce certain AMD‐like features in the RPE. For this purpose, lyophilized dECM‐BrM was pepsin‐digested and the resulting mixture was used as a coating substrate for primary porcine RPE (pRPE) culture in Transwell filters. Primary pRPE were grown in aged and young dECM‐BrM‐coated inserts for up to 100 days. Interestingly, both aged and young dECM‐BrM enabled pRPE survival and polarization. Confocal microscopy revealed defined ZO‐1 expression, characteristic cobblestone morphology, and pigmentation of pRPEs (Figure 4A,B). TEER values reached 300 Ohm*cm^2^ after 3 weeks of culture (Figure 4C). Of note, after 60–70 days TEER was significantly lower in pRPE grown in aged dECM‐BrM compared to those grown in young dECM‐BrM (*P* < 0.05).

To test the proinflammatory phenotype of pRPE cells, a range of secreted cytokines were measured in the supernatant of apical and basal compartments. Polarized secretion of IL‐33 increased over time and was significantly higher after 80 days of culture in pRPE grown in aged dECM‐BrM compared to those grown in young dECM‐BrM (*p* < 0.05) (Figure 4D). Secreted IL‐17 tended to decrease over time with no effect of the coating substrate (Figure 4E). IL‐18, another IL‐1‐related cytokine, decreased over time, particularly in pRPE grown in young dECM‐BrM (*p* < 0.05) (Figure 4F). In turn, no differences were observed in secreted IL‐8 and TNF‐α at different time points and with different coating substrates (Figure 4G,H).

**FIGURE 5:**
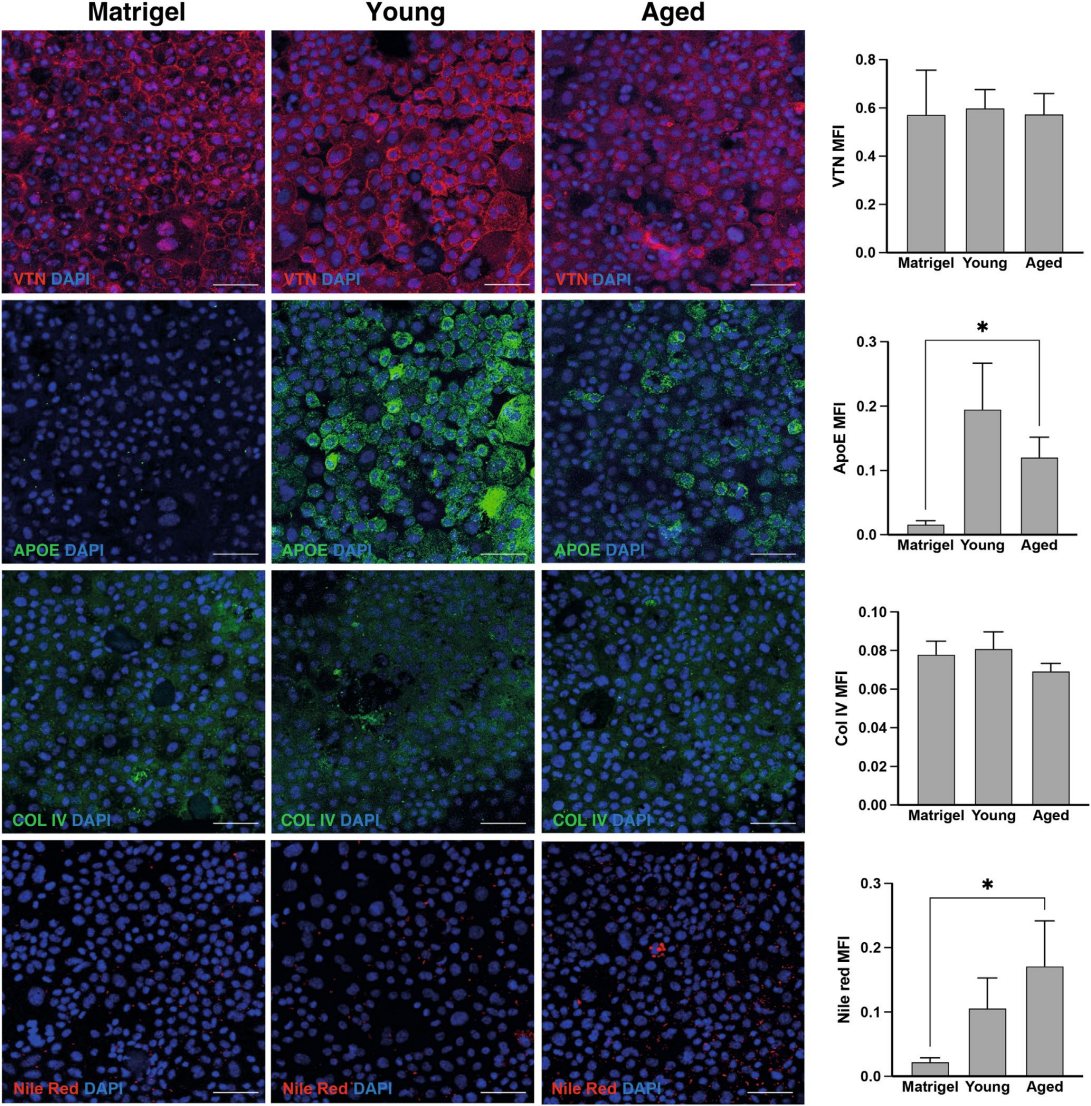
Expression of drusen components in pRPE cells grown on aged dECM‐BrM. Staining of vitronectin, Apolipoprotein E, Collagen IV, and Nile red in pRPE monolayers grown on Matrigel, young dECM‐BrM, and aged dECM‐BrM. Scale bar = 50 μm. Images shown are representative of three independent experiments. Data are shown as mean ± SEM. *p < 0.05 calculated using one‐way ANOVA followed by Tukey's posthoc analysis.

**FIGURE 6:**
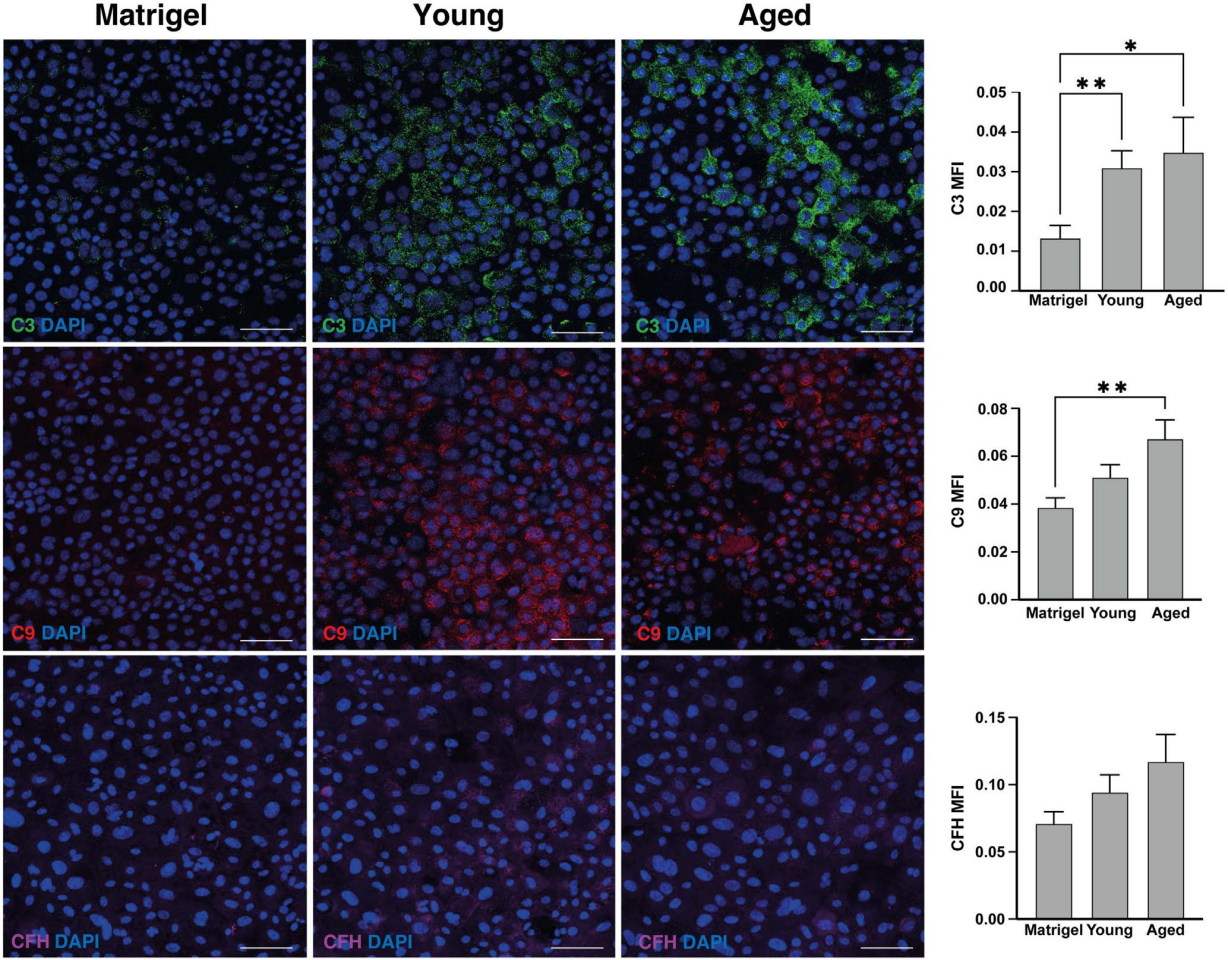
Expression of complement components in pRPE cells grown on aged dECM‐BrM. Immunostaining of complement components C3, C9, and CFH in pRPE monolayers grown on Matrigel, young dECM‐BrM, and aged dECM‐BrM. Scale bar = 50 μm. Images shown are representative of three independent experiments. Data are shown as mean ± SEM. Statistical analysis was performed using one‐way ANOVA followed by Tukey's post hoc analysis, *p < 0.05, **p < 0.01.

Immunofluorescence analysis revealed the presence of several drusen components in pRPE cells grown on aged dECM‐BrM (Figure 5). Vitronectin was highly abundant in pRPE cells after 65 days of culture. Interestingly, Apolipoprotein E expression was significantly higher in pRPE grown on aged dECM‐BrM compared to pRPE cells grown in Matrigel (*p* < 0.05), where it was nearly absent (Figure 5). Collagen IV was similarly detected on pRPE cells grown either on Matrigel or dECM‐BrM with a heterogeneous distribution (Figure 5). To determine the presence of polar and neutral lipids, including esterified cholesterol, samples were stained with Nile red. Notably, lipid droplets were significantly more abundant on pRPE cells grown on aged dECM‐BrM compared to Matrigel (*p* < 0.05) (Figure 5). Complement protein C3 and C9 were detected only at low levels in pRPE cells grown on Matrigel, while their expression was significantly higher in cells grown on aged dECM‐BrM (*p* < 0.05). Notably, C3 expression in pRPE cells grown on young dECM‐BrM was also significantly higher than in Matrigel (*p* < 0.01). No statistically significant differences were observed in CFH expression (Figure 6).

**FIGURE 7:**
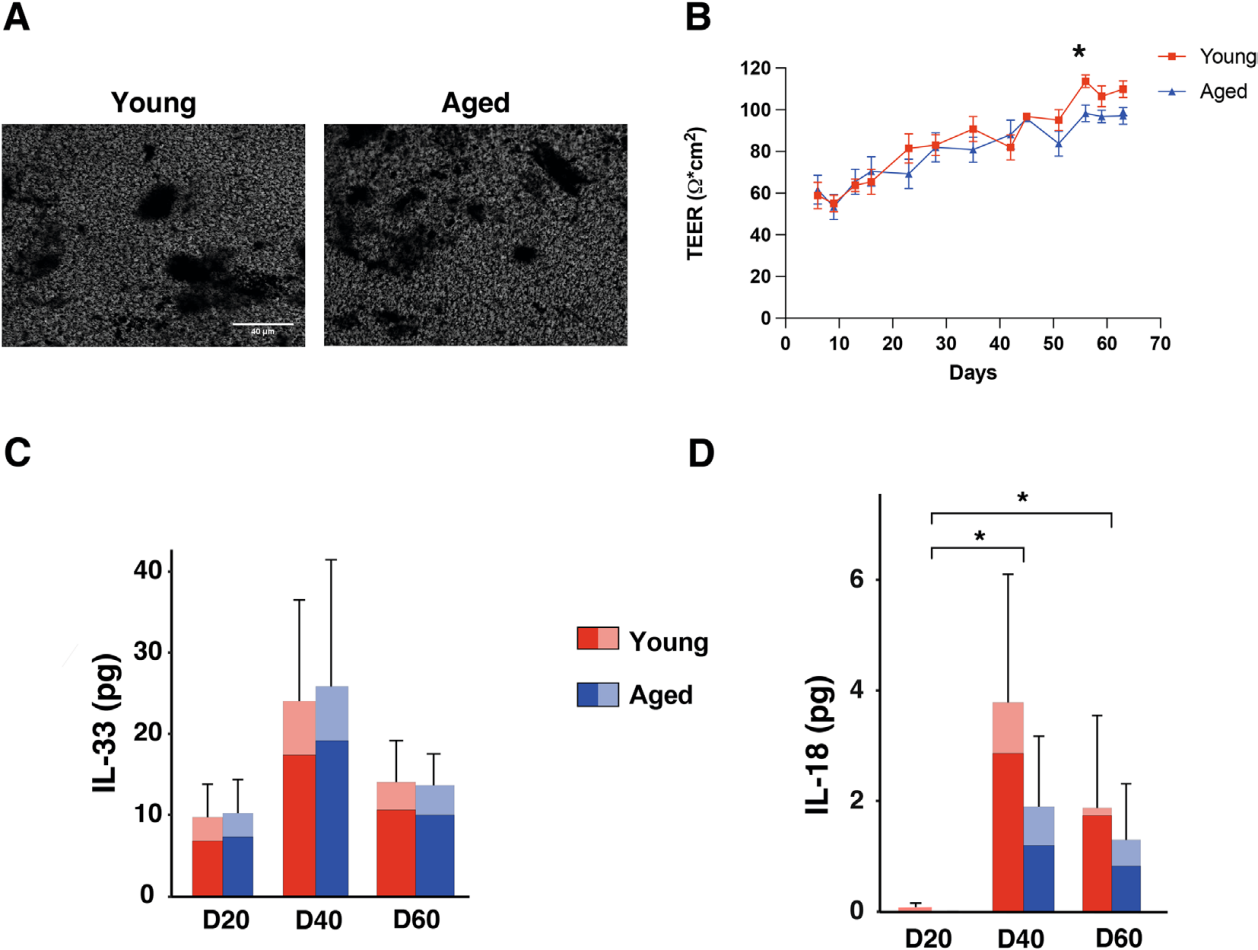
Effect of aged dECM‐BrM coating substrate on hRPE function and phenotype. (A) Confocal microscopy images of transmitted light in hRPE cells grown on Transwell inserts for 65 days on aged and young dECM‐BrM. (B) Transepithelial electrical resistance (TEER) of primary hRPE cells grown on Transwell inserts coated with aged or young dECM‐BrM for 65 days. Data are shown as mean ± SEM, *n* = 3. Statistical analysis was performed by Student's *t*‐test. *p < 0.05. Secretion of IL‐33 (C) and IL‐18 (D) by hRPE cells grown on dECM‐BrM‐coated transwell inserts. Light color indicates basolateral secretion whereas dark color reflects apical secretion Data are shown as mean ± SEM, *n* = 4. *p < 0.05 calculated using Mann–Whitney's test for comparisons between two independent conditions, and Kruskal‐Wallis followed by post hoc Dunn's test for comparisons across multiple time points.

**FIGURE 8:**
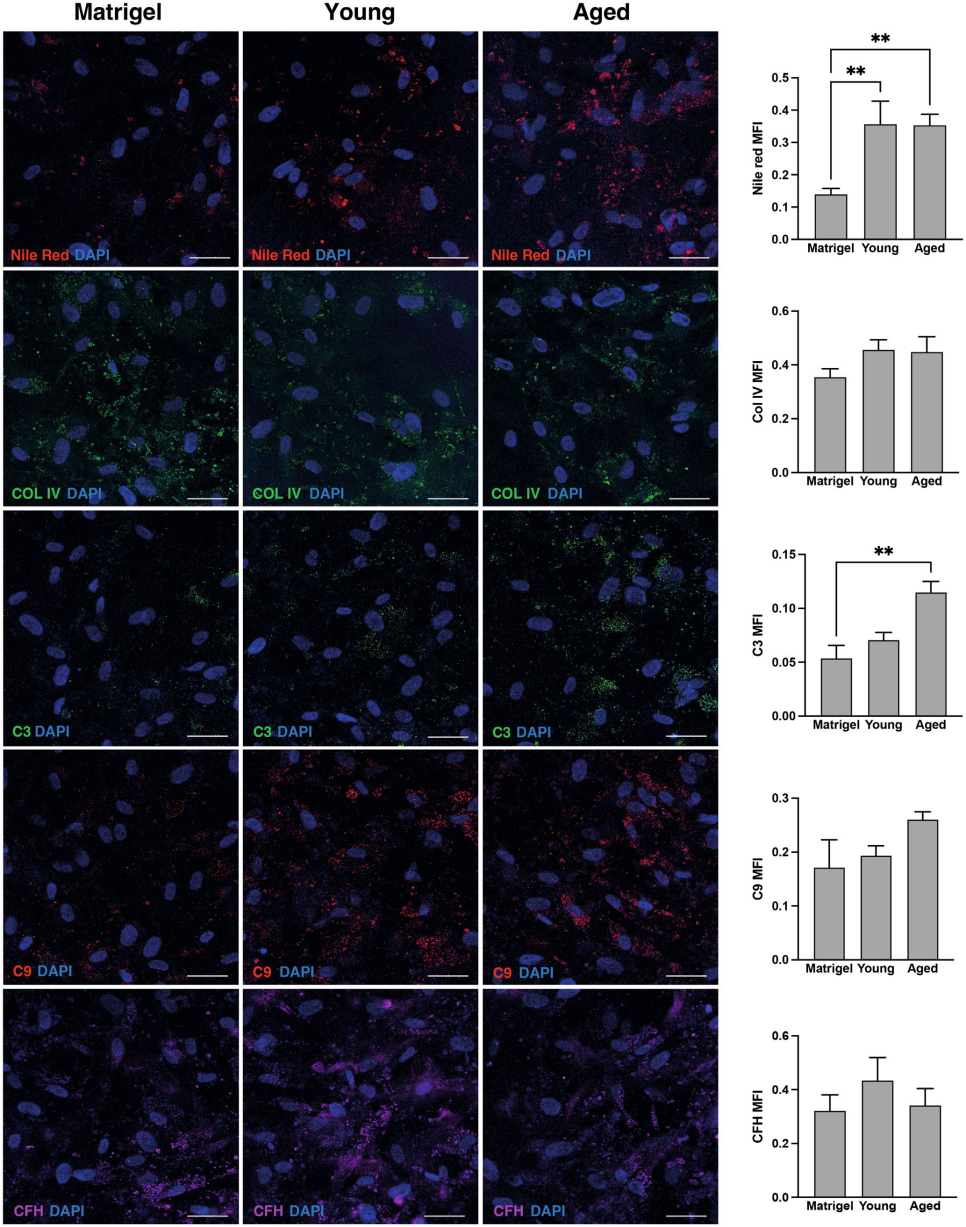
Expression of drusen components in hRPE cells grown on dECM‐BrM. Staining of Nile red, Collagen IV, and complement components C3, C9, and CFH in hRPE monolayers grown on Matrigel, young dECM‐BrM, and aged dECM‐BrM. Scale bar = 50 μm. Images shown are representative of two independent experiments. Data are shown as mean ± SEM. Statistical analysis was performed by One‐Way ANOVA followed by Tukey's posthoc analysis, **p < 0.01.

Human RPE cells obtained from elderly donors grown on dECM‐BrM were also pigmented (Figure 7A) but less polarized than pRPE as seen by TEER levels that did not exceed 110 Ohm*cm^2^ (Figure 7B). Similar to pRPE cells, primary hRPE cells grown on aged dECM‐BrM showed significantly reduced levels of TEER after 50 days of culture (*p* < 0.05) compared to those grown on young dECM‐BrM (Figure 7B). Until Day 60, no differences were observed on IL‐33 secretion in hRPE cells and secreted IL‐18 significantly increased from day 20 to Day 40, which was then reduced again at Day 60 (Figure 7). Similar to pRPE cells, C3 and lipid droplets were significantly higher in hRPE cells grown on aged dECM‐BrM (*p* < 0.05). However, no statistically significant differences were observed in collagen IV, C9, and CFH (Figure 8).

## 4 Discussion

The shortage of efficient therapeutic options for the treatment of advanced AMD underscores the need for adequate physiologically relevant AMD models to understand the mechanisms underlying AMD and allow testing of novel therapeutic agents. In the current study, we deepened the current knowledge related to age‐associated changes of the proteomic profile of dECM‐BrM. Leveraging such changes, we developed a BrM‐like coating substrate based on dECM‐BrM from elderly donors to build an AMD in vitro model.

Decellularized‐based scaffolds have emerged in the last decade as an excellent strategy for tissue modeling as they ideally preserve the 3D structure and biochemical composition of the native tissue, thus providing site‐specific cues. To effectively remove the cellular component of the RPE/BrM/choroid tissue we combined physical, enzymatic, and chemical methods based on the optimization of our previous studies (Molins et al. 2021). Our decellularization protocol guarantees cell removal, fundamental to mitigate alloresponses while preserving the ECM. Nevertheless, it is recognized that decellularization strategies carry certain ECM disruption and we also observed partial disruption and loss of elastin fibers and soluble collagen.

Using a proteomics approach, we characterized the composition of human dECM‐BrM. We confirmed that despite decellularization, both young and aged dECM‐BrM preserved ECM components of the BrM such as collagens, laminins, elastin, and proteoglycans. These matrix components confer structural support and key biochemical cues for RPE adhesion, maturation, and differentiation, which are required for adequate barrier function (Naba et al. 2016; Hynes 2009). As expected, and validating our findings, most of the identified proteins have been previously identified in the BrM proteome. Notably, we identified distinctive proteins of the BrM such as TIMP3, HTRA1, and EFEMP1. Based on the Matrisome project, most of the identified matrisome proteins in dECM‐BrM were classified as core matrisome (collagens, ECM glycoproteins, and ECM proteoglycans) and ECM regulators (matrisome‐associated). We identified the main collagen types present in the 5 layers forming the BrM (Types I, III, IV, V, and VI) and several proteoglycans were also identified, being the small leucine‐rich proteoglycans (SLRPs) biglycan, lumican, and prolargin particularly abundant. Both young and aged dECM‐BrM showed a similar matrisome profile with young dECM‐BrM specifically enriched in ECM proteoglycans. The matrisome profile of dECM‐BrM was consistent with the matrisome profile of decellularized murine uvea previously reported elsewhere (Li et al. 2022). About half of the identified proteins in both young and aged dECM‐BrM were classified as non‐matrisome proteins by the Matrisome Project. These mostly consisted of apolipoproteins, clusterin, serum amyloid P, complement components, and other proteins abundant in the BrM and in drusenoid deposits (Yuan et al. 2010; Crabb et al. 2002). On the other hand, besides the identification of several keratins that may be the result of sample contamination during experimental manipulation, several histones were also identified, especially in young dECM‐BrM. Histones are highly positively charged molecules that could have been retained by anionic glycosaminoglycans during the decellularization process. Histones may behave as damage‐associated molecular patterns and its potential contribution to the ECM‐specific biochemical cues, although unlikely due to its limited bioavailability, cannot be completely excluded. Our findings are in line with a previous study of Chirco et al. who prepared decellularized choroids to use as scaffolds for choroidal endothelial cell repopulation (Chirco et al. 2017). In their study, the authors also performed a proteomic analysis by LC–MS of native and decellularized BrM‐choroids and more than 90% of their identified proteins were also identified in our proteomic analysis. Highly relevant proteins for AMD including complement components (C9 and C3), VTN, APOE, APCS, and TIMP3 were identified as highly present in dECM‐BrM in both studies. Indeed, GO enrichment analysis identified “ECM structural constituent”, “ECM organization”, and “complement activation” among the most significantly enriched GO categories in both studies.

The fact that the dECM‐BrM proteome was significantly enriched in biological processes related to AMD such as angiogenesis, innate immune response, complement activation and inflammatory response underscores the key role that the ECM (dECM‐BrM) may play in AMD development. Of note, the quantitative analysis revealed that 37 proteins were significantly upregulated in aged dECM‐BrM compared to young dECM‐BrM. Among them, metalloproteinase inhibitor TIMP3, C7, C8, clusterin, and BASP1 have been found to be overexpressed in the BrM of AMD patients compared to healthy aged donors in a previous proteomics study (Yuan et al. 2010). Other upregulated proteins like EFEMP1, HTRA1, and APOE have also been related to AMD (Fernandez‐Godino et al. 2018; Lin et al. 2018; Klaver et al. 1998). Interestingly, we found that these proteins were overexpressed in aged donors with no history of advanced AMD. Notably, we identified 7 proteins in aged dECM‐BrM that were not detected in young dECM‐BrM: DEFA4, GOT2, ANXA4, F9, SFRP5, IGKV3‐20, and FABP5. DEFA4 is an antimicrobial peptide of the innate immune system, and although it has not been previously related to aged BrM or AMD, 2 members of the same alpha‐defensin family, DEFA1 and DEFA3, have been identified in the BrM of aged donors with AMD (Yuan et al. 2010). While the involvement of GOT2, ANXA4, F9, and IGKV3‐20 in retinal homeostasis has yet to be described, the protein SFRP5, an anti‐inflammatory adipokine, contributes to retinal development (Holly et al. 2014) and FABP5 plays a critical role in lipid metabolism in the RPE (Wu et al. 2010). C1QTNF5, a membrane associated protein expressed in the RPE with mutations associated with late‐onset retinal degeneration (Fernández and Carr 2025), was 20‐fold overexpressed in aged dECM‐BrM.

Overall protein content in aged dECM‐BrM was higher than in young dECM‐BrM and even the macroscopic appearance showed a higher consistency that could be the result of increased protein accumulation and cross‐linking in aged BrM. Indeed, quantitative proteomics revealed 37 proteins significantly enhanced in aged dECM‐BrM while only 12 proteins were significantly enhanced in young dECM‐BrM. Gene Ontology enrichment analysis provided a global understanding of the main biological processes and molecular functions related to differentially expressed proteins. Interestingly, KEGG pathway analysis showed that differentially expressed proteins were significantly enriched in the complement cascade, a key mechanism of AMD development and progression (Romero‐vazquez et al. 2021). Given the aged‐associated reduction in BrM permeability and the source of BrM components, the observed changes in aged dECM‐BrM proteome are likely to be the result of altered RPE protein secretion. Paraoan et al. identified 30 secretory proteins within the 200 most abundantly expressed transcripts in the RPE/choroid (Paraoan et al. 2020). We identified 20 out of these 30 proteins in our dECM/BrM samples (TIMP3, PSAP, CLU, SERPIN, CP, PEDF, LAMB2, A2M, NMB, DCN, ALDOA, APOD, BGN, CTSB, CTSD, MFAP4, C1QTNF5, COL1A2, PRELP, TF). Notably, among these RPE secreted proteins, TIMP3, CLU, LAMB2, A2M, CTSD, and C1QTNF5 were differentially expressed in aged dECM‐BrM. Other authors have evaluated the impact of AMD or AMD‐like conditions in RPE secretome, showing increased secretion of drusen‐associated proteins (CLU, APOE, VTN), complement‐related proteins (C4a, CFI, CFB, C3, C1QTNF5), and proteins related to ECM remodeling (TIMP3, LUM, MMP2, FN, GAL3) in AMD (Flores‐Bellver et al. 2021; Meyer et al. 2019; An et al. 2006). Among the 49 differentially expressed proteins in aged dECM‐BrM, 18 have been shown to be secreted by RPE cells and 9 of them (TIMP3, APOE, CLU, COL10A1, FBN1, C1QTNF5, SOSTDC1, EFEMP1, and LAMB2) are among the 50 most abundant proteins in the dECM‐BrM samples highlighting the impact of the RPE secretome in aged BrM remodeling.

The use of aged dECM‐BrM as a coating substrate for pRPE culture allowed us to reproduce certain features associated with AMD. RPE cells have been previously grown ex vivo on human aged BrM showing that BrM aging reduces adhesion, proliferation, and phagocytosis of photoreceptor outer segments in RPE cells (Sun et al. 2007; Tezel et al. 2004). Nevertheless, access to human tissue donors is scarce and needs to be used fresh, hindering the applicability of such ex vivo models. In our approach we also used human tissue but the preparation of the coating substrate containing dECM‐BrM required less starting material. From a pool of 3 samples, we prepared 1.5 mg of young dECM‐BrM and 2 mg of aged dECM‐BrM and given that we used the coating solution at a concentration of 100 μg/mL, we were able to seed 75 wells (12 mm) with young dECM‐BrM and 100 with aged dECM‐BrM. In addition, the prepared biomaterial could be stored at −20°C or 4°C facilitating its practical use. Alternatively, a synthetic coating substrate based on a specific cocktail combination of certain overexpressed identified proteins could also serve to model the aged BrM.

The coating substrate based on dECM‐BrM allowed RPE survival, polarization, and pigmentation that were maintained for at least 100 days. Interestingly, after 60 days of culture, TEER values of pRPE grown on aged dECM‐BrM were significantly lower than those grown on young dECM‐BrM, suggestive of a lower barrier function. The analysis of secreted proinflammatory cytokines also revealed significant differences in IL‐33. IL‐33 is a member of the IL‐1 superfamily that drives strong proinflammatory signaling acting as an alarmin released upon cell injury (Cayrol and Girard 2022). In the eye, IL‐33 is mainly expressed in glial, RPE, and endothelial cells. In patients with AMD, increased IL‐33 vitreous levels and IL‐33+ Müller cells in areas of retinal atrophy have been observed (Xi et al. 2016). In addition, retinal stress activates the IL‐33 pathway resulting in increased release of proinflammatory cytokines and cell recruitment in the outer retina. Yet, IL‐33 also regulates tissue remodeling by attenuating wound‐healing responses and inhibiting choroidal neovascularization (Theodoropoulou et al. 2017). Indeed, both therapies enhancing and inhibiting IL‐33 signaling have been proposed for AMD (Clare et al. 2020; Pearlman et al. 2025). We also observed a progressive decrease in IL‐18 secretion, another cytokine from the IL‐1 superfamily that has been associated with AMD (Schloesser et al. 2024). The NLRP3 inflammasome is activated in AMD in response to drusen components leading to the production of IL‐1β and IL‐18 conferring protection against progression to neovascular AMD because IL‐18 seems to regulate angiogenesis in the choroid (Doyle et al. 2014, 2012). Given the decrease in IL‐18 and that no differences were observed in TNF‐α and IL‐8 secretion, the increase in polarized IL‐33 secretion in pRPE cells grown on aged dECM‐BrM may reflect a stress‐related response rather than a fully specific AMD‐related signature. A deeper understanding of how age‐related changes in the BrM regulate IL‐33 and IL‐18 in the RPE could help to decipher the triggering factors of the transition from healthy aging to chronic stress and eventually early AMD.

To maximize translationality, in vitro AMD models should use human RPE. However, the scarcity and cost of human globes for primary human RPE culture hinders its use (Bharti et al. 2022). In addition, in the last years, the widely used human ARPE‐19 cell line has also shown several important differences from human primary RPE cells, showing an abnormal karyotype and an altered transcriptomic and proteomic profile that make them a poor physiologically relevant model of native RPE (Bharti et al. 2022). Alternatively, we and others have used primary porcine RPE cells, as they are accessible and inexpensive, to model AMD in vitro (Shaw et al. 2024; Romero‐Vázquez et al. 2020). Primary porcine RPE cultures grown on Transwell inserts show a polarized phenotype and even develop drusen‐like deposits and complement activation after 20 weeks of culture (Shaw et al. 2024; Johnson et al. 2011). Recently, Shaw and collaborators developed a drusen‐in‐a‐dish model based on porcine pRPE cells grown on Transwell inserts comparable to human drusen (Shaw et al. 2025). By using immunohistochemistry and imaging mass spectrometry (IMS), authors performed a detailed characterization of the composition of sub‐RPE deposits of pRPE monolayers grown for 20 weeks. In our study, we used confocal microscopy to determine the expression of several drusen components in pRPE monolayers grown on dECM‐BrM. Interestingly, Apolipoprotein E, C3, C9, and lipid droplets were highly abundant in pRPE cells grown on aged dECM‐BrM already after 9 weeks of culture (65 days), compared to RPE cells grown on Matrigel, suggesting that dECM‐BrM may provide specific cues to induce an AMD‐like environment earlier than standard coating substrates. Although certain intracellular staining cannot be excluded, the detected immunofluorescence expression represents extracellular accumulation as staining was performed in non‐permeabilized RPE. Nevertheless, a more comprehensive characterization of the composition and localization of such deposits in RPE cells grown on aged dECM‐BrM would help to elucidate whether they represent structural drusen‐like deposits, highlighting the suitability of aged dECM‐BrM to model AMD. To increase the translational value of our work, we replicated some experiments with human primary RPE cells obtained from adult donors. Similar to porcine cells, hRPE cells grown on aged dECM‐BrM showed decreased TEER after 50 days of culture, with increased deposition of the complement component C3 and lipid droplets, demonstrating that aged dECM‐BrM provides a unique scenario that induces certain features suggestive of an AMD‐like phenotype. Yet, experiments with human RPE should be interpreted with caution as they were preliminary in terms of duration and characterization. A deeper characterization of the hRPE phenotype and the sub‐RPE deposits of hRPE cells grown on dECM‐BrM for longer periods are warranted.

Our study carries certain limitations. Firstly, although aged human donors did not have AMD diagnosis and they were all below 70 years old, we cannot exclude the presence of incipient AMD with small drusen in the RPE/BrM tissue and therefore the differences observed in the proteomic profile of aged and young dECM‐BrM might also capture the presence of early disease. Although donors had no history of retinal disease, other baseline systemic comorbidities might be differentially present in donor subjects, thus potentially influencing oBRB properties and composition and the observed proteomic differences might also partially reflect systemic comorbidities in addition to age‐related changes. The use of RPE derived from human inducible pluripotent stem cells (hiPSCs), especially if carrying genetic risk variants associated with AMD, would provide higher translational application of our model. Despite hiPSC‐derived RPE may not properly reflect the phenotype of an aged RPE, they can reproduce AMD features if cultured for long periods. Overall, we demonstrated the suitability of a coating substrate based on dECM‐BrM for RPE culture and that aged dECM‐BrM confers a specific microenvironment that induces certain features associated with AMD such as reduced barrier function, increased secretion of IL‐33, and increased deposition of Apolipoprotein E, complement factor C3, C9, and lipid droplets. Nevertheless, a more comprehensive study and comparison of RPE grown on young versus aged dECM‐BrM by transcriptomic analysis and/or IMS would help to elucidate how aged ECM of the oBRB modulates RPE behavior.

## 5 Conclusion

In the present study, we provided a deeper understanding of the age‐associated changes in the proteome of dECM‐BrM that can help elucidate the switches that promote the transition from healthy aging to AMD. Building on this hypothesis, we developed a biomaterial based on aged dECM‐BrM that was then used as a coating substrate for RPE culture for AMD modeling. Indeed, RPE monolayers grown on aged dECM‐BrM showed certain features consistent with an AMD‐like phenotype such as reduced TEER, altered IL‐33 production, and increased Apolipoprotein E, C3, C9, and lipid deposition. Although a deeper characterization of the impact of aged dECM‐BrM on RPE function and phenotype is warranted, our study offers a promising in vitro platform to study AMD.

## Author Contributions

**Blanca Molins:** conceptualization, investigation, methodology, formal analysis, funding acquisition, supervision, writing – original draft, visualization. **Mercedes Soledad Nabaes Jodar:** investigation, methodology, visualization, writing – review and editing. **Eliandre de Oliveira:** investigation, methodology, formal analysis, resources, data curation, writing – review and editing. **Josep Maria Estanyol:** formal analysis, data curation, validation, writing – review and editing. **Esther Peña:** investigation, resources, writing – review and editing. **Jaione Bezunartea:** investigation, resources, writing – review and editing. **María Hernández:** investigation, resources, writing – review and editing. **Marc Figueras‐Roca:** investigation, funding acquisition, writing – review and editing. **Alfredo Adan:** funding acquisition, resources, supervision, writing – review and editing.

## Funding

The author(s) declare that financial support was received for the research and/or publication of this article. This work has been funded by Instituto de Salud Carlos III (ISCIII) through the project PI22/00782 and co‐funded by the European Union. This work was supported by Novartis Pharmaceuticals through an Investigator‐Initiated Study (IIS) grant. The sponsor had no involvement in study conceptualization, data collection and analysis, decision to publish, or preparation of the manuscript.

## Conflicts of Interest

The authors declare no conflicts of interest.

## Supporting information


**Table S1:** List of proteins identified by LC–MS/MS in human dECM‐BrM samples.

**Table S2:** Gene Ontology Biological Process (GOBP) enrichement analysis of the dECM‐BrM proteome.

**Table S3:** Gene Ontology Cellular Component (CC) enrichment analysis of the dECM‐BrM proteome.

**Table S4:** Gene Ontology Molecular Function (MF) enrichement analysis of the dECM‐BrM proteome.

**Table S5:** Gene Ontology KEGG pathways enrichment analysis of the dECM‐BrM proteome.

## Data Availability

The data that supports the findings of this study are available in the Supporting Information of this article.
